# Novel cutaneous adverse effects of the increasingly prevalent glucagon-like peptide 1 agonists

**DOI:** 10.1093/skinhd/vzag090

**Published:** 2026-07-06

**Authors:** Luke Carson, Mark Eisner, Caroline Launay, Suzy Leech, Sophie Weatherhead

**Affiliations:** Translational and Clinical Research Institute, Newcastle University, Newcastle-upon-Tyne, UK; Department of Dermatology, Royal Victoria Infirmary, Newcastle upon Tyne, UK; Translational and Clinical Research Institute, Newcastle University, Newcastle-upon-Tyne, UK; Department of Dermatology, Royal Victoria Infirmary, Newcastle upon Tyne, UK; Department of Cellular Pathology, Royal Victoria Infirmary, Newcastle upon Tyne, UK; Department of Dermatology, Royal Victoria Infirmary, Newcastle upon Tyne, UK; Department of Dermatology, Royal Victoria Infirmary, Newcastle upon Tyne, UK

## Abstract

Prescribing of the glucagon-like peptide 1 receptor agonists (GLP-1RA) is rapidly increasing as their efficacy for multiple indications becomes apparent. A better understanding of their cutaneous adverse effect profile is crucial, particularly as they are suggested for use in inflammatory dermatoses. We describe two patients who developed novel cutaneous reactions associated with initiation of a GLP-1RA. Patient 1 developed multiple tender subcutaneous nodules on the trunk and limbs 4 weeks after the initiation of semaglutide. Histology demonstrated a noncaseating granulomatous dermatitis, and serum ­angiotensin-converting enzyme was elevated. A diagnosis of cutaneous (and likely drug-induced) sarcoidosis was made once systemic involvement had been excluded. On cessation of semaglutide, all lesions resolved without additional treatment. The Naranjo score indicated a probable drug reaction. Patient 2 presented with a recurrence of solar urticaria, confirmed on phototesting, 5 days after commencement of liraglutide, having previously been in remission for over 20 years. Her course was recalcitrant, despite cessation of liraglutide and a broad range of therapies. The Naranjo score suggested a probable association. These cases represent the first descriptions of cutaneous sarcoidosis and solar urticaria associated with GLP-1RA ­therapy. While further work is required to define possible underlying mechanisms, we report these cases due to the convincing temporal relationship, high Naranjo scores and the large patient population now currently exposed to this drug class. Dermatologists should be aware of the possible cutaneous adverse or exacerbatory effects, as well as the potential significant benefits, of these increasingly commonly used medications.

## Teaser

The glucagon-like peptide 1 receptor agonists are becoming ever more prevalent, with their efficacy described for multiple indications. As more patients take these medications, a knowledge of potential side-effects is critical. We report the first descriptions of solar urticaria and cutaneous sarcoidosis following the use of this medication class.

What is already known about this topic?

- The cutaneous adverse effects of glucagon-like peptide 1 receptor agonists were reviewed in 2024.
- No reports of solar urticaria or cutaneous sarcoidosis were present at the time of this review.

What does this study add?

- We report the first descriptions of solar urticaria and cutaneous sarcoidosis temporally associated with the use of this medication class.

The glucagon-like peptide 1 receptor agonists (GLP-1RA) semaglutide and liraglutide are National Institute for Health and Care Excellence-approved for type 2 diabetes mellitus and weight loss management in England. Some 4.2 million people will be eligible for semaglutide for weight management in England by 2027,^1^ and such medications can currently be obtained from private providers. GLP-1RA have been shown to be beneficial for treating hidradenitis suppurativa^2^ and psoriasis.^3^ As more patients take these medications, an understanding and recognition of any cutaneous adverse effects is becoming increasingly important. We present the first reported cases of solar urticaria (SU) and cutaneous sarcoidosis associated with the use of GLP-1RA.

Patient 1 was a woman in her sixties referred by her general practitioner with a 3-month history of nodules forming on her trunk and limbs. Four weeks prior to the lesions developing she had commenced semaglutide for weight control; no other new prescribed or over-the-counter medications, other remedies, recreational drug use or other confounders such as infections were reported. After the third injection she had developed lesions felt by the general practitioner to be consistent with erythema nodosum. After the fourth injection she developed further multiple nodules and stopped the injections at that point. She had multiple mildly tender, firm, flesh-coloured nodules on the arms, legs and abdomen ranging in size from 2 to 6 cm (Figure 1a). They were independent of injection sites. A punch biopsy taken at time of presentation from lesions on the forearm (Figure 1a) demonstrated noncaseating granulomatous dermatitis (Figure 1b). All stains for infection were negative. Her clinicopathological diagnosis was the Darier-Roussy form of sarcoidosis. An extractable nuclear antigens screen was negative. Serum angiotensin-­converting enzyme was raised (100 unit L^–1^), although spirometry demonstrated a restrictive pattern [forced expiratory volume in 1 s (FEV1) 1.75 L, 77% predicted; forced vital capacity (FVC) 2.12 L, 74% predicted], the gas transfer coefficient was elevated (KCO 1.98, 126% predicted). Chest X-ray was normal. This pattern is characteristic of extrapulmonary restriction and pulmonary sarcoidosis was excluded on review in a tertiary interstitial lung disease clinic. Five months after stopping the semaglutide the lesions had all resolved, with no other intervention. The Naranjo Adverse Drug Reaction Probability Scale score was 7 (probable).^4^ (See Appendix 1.)

**Figure 1:**
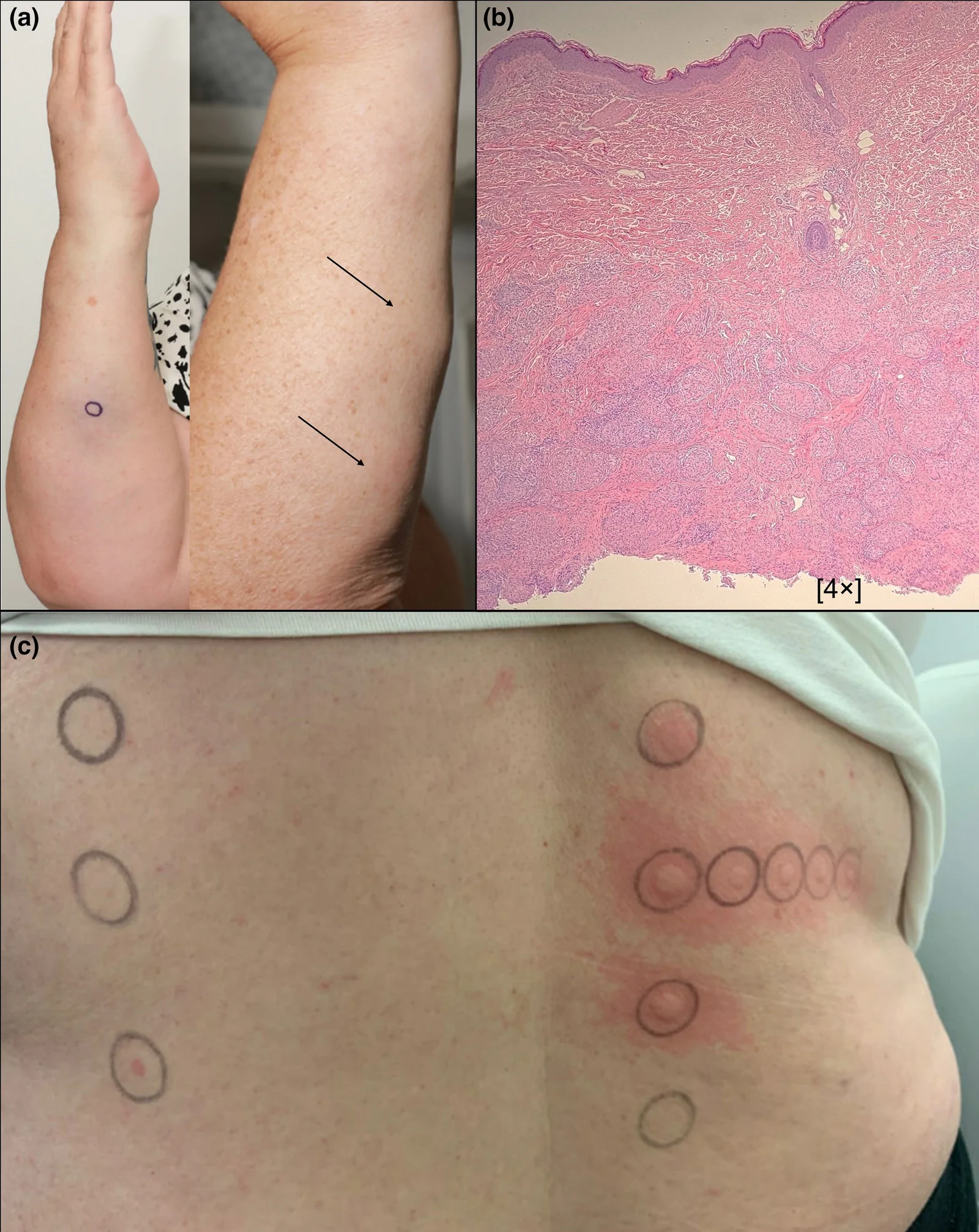
(a) Clinical images of patient 1 at presentation, with multiple firm, flesh-coloured nodules and biopsy site pictured. (b) Histological appearance (patient 1) at ×4 magnification, demonstrating a noncaseating granulomatous dermatitis (haematoxylin and eosin). (c) Clinical image from patient 2 demonstrating florid urticarial response on monochromator phototesting. Solar urticaria was confirmed at wavelengths of 450 and 500 nm (positive reaction down to the minimum tested dose of 2.5 J cm^–2^ at 500 nm). Solar urticaria could not be induced at 350 or 550 nm.

Patient 2 was a woman in her sixties who had been under the care of dermatology for SU 24 years previously, which had been in complete remission following treatment with psoralen + UVA. She re-presented after 20 years of clearance with a recurrence of SU, which had started 5 days following the commencement of liraglutide for weight loss. As in our first case, no other potential causes or confounders were noted. SU was confirmed on monochromator phototesting at wavelengths of 450 and 500 nm (positive reaction down to the minimum tested dose of 2.5 J cm^–2^ at 500 nm), undertaken 2 weeks following presentation to clinic. SU could not be induced at 350 or 550 nm. Her total IgE was raised at 487 IU mL^–1^ (normal range 0–100). Her disease course remained recalcitrant despite cessation of liraglutide, alongside treatment with fexofenadine, loratadine, sodium cromoglicate, famotidine, montelukast and omalizumab. Due to a lack of response to omalizumab, a further course of PUVA was trialled, which had previously been useful, and subsequently methotrexate, ciclosporin, Heliocare capsules and azathioprine, all of which were not ­tolerated or failed to control her condition. Unfortunately, an application for compassionate funding for dupilumab to be used off-licence was rejected. A further course of PUVA is currently being trialled. Her clinical course was unusual in its initial recalcitrance to treatments, including omalizumab at standard dosing of 300 mg once monthly, and subsequent PUVA. Following national discussion she was trialled on an increased dose of omalizumab (600 mg once monthly), with which she has achieved an excellent response. The Naranjo Adverse Drug Reaction Probability Scale score was 5 (probable).^4^

To our knowledge, these are the first cases of cutaneous sarcoidosis or SU temporally associated with the use of GLP-1RA (Table S1; see Supporting Information). Interestingly, a recent case report described erythema nodosum after the initiation of semaglutide.^5^ Sarcoidosis has been reported in the UK’s Yellow Card scheme as an adverse effect of semaglutide.^6^ Neither SU nor sarcoidosis were described in a recently published review of the ­literature.^7^ Medications typically associated with drug-induced sarcoidosis are immunomodulatory.^8^ GLP-1 has been demonstrated to act on macrophages, which are central to sarcoidosis, causing a switch towards M2 polarization (via the cAMP–PKA–STAT3 pathway),^9^ a state associated with granuloma formation in sarcoidosis.^10^ Regulatory T cells have also been shown to be upregulated by GLP-1 and its analogues,^9^ which is also demonstrated in a few sarcoidosis studies.^10^ Significant weight loss may also be responsible for a shifting immune state: obesity is linked to M1 macrophage polarization for example.^10^ While the mechanism for SU is less well understood, early research suggests lipid metabolism may play an important role in the pathogenesis.^11^ The potential for GLP-1RA to interact in this pathway is an open question for future work. Recent literature has also highlighted the role of GLP-1RA in modulating angiogenesis, which is known to have a role in chronic inflammatory dermatoses including urticaria.^12,13^

These cases warrant reporting due to the convincing temporal relationship, a high Naranjo score (although not a specific scoring system for immune-mediated skin reactions) and the large patient population now exposed to this drug class. Further studies are necessary to elucidate the pathophysiology of these potential adverse or exacerbating events. Dermatologists should be aware of the cutaneous adverse effects, as well as the potential significant benefits, of these increasingly commonly used medications.

## Supplementary Material

vzag090_Supplementary_Data

## Data Availability

No new data were generated or analysed in support of this research.

## Appendix 1 Naranjo scoring^4^

**Table:**

Question	Yes	No	Do not know	Case 1 score	Case 2 score
1. Are there previous conclusive reports on this reaction?	+1	0	0	0	0
2. Did the adverse event appear after the suspected drug was administered?	+2	−1	0	+2	+2
3. Did the adverse event improve when the drug was discontinued or a specific antagonist was administered?	+1	0	0	+1	0
4. Did the adverse event reappear when the drug was readministered?	+2	−1	0	0	0
5. Are there alternative causes that could on their own have caused the reaction?	−1	+2	0	+2	+2
6. Did the reaction reappear when a placebo was given?	−1	+1	0	0	0
7. Was the drug detected in blood or other fluids in concentrations known to be toxic?	+1	0	0	0	0
8. Was the reaction more severe when the dose was increased or less severe when the dose was decreased?	+1	0	0	0	0
9. Did the patient have a similar reaction to the same or similar drugs in any previous exposure?	+1	0	0	+1	0
10. Was the adverse event confirmed by any objective evidence?	+1	0	0	+1	+1
Total score				7	5
