# Supplementary material for: Novel cutaneous adverse effects of the increasingly prevalent glucagon-like peptide 1 agonists
Source: Skin Health Dis. 2026 Jul 6;6(5):808–11. doi: 10.1093/skinhd/vzag090 (PMC13623448; doi:10.1093/skinhd/vzag090)
Supplement: Supplementary file 1 [file vzag090_supplementary_data.docx]

| **Case 1** | |
| --- | --- |
| Suspected trigger | Semaglutide (Wegovy), 0.25mg once weekly |
| Time from medication commencement to disease onset | 4 weeks |
| Concomitant medications | Letrozole 2.5mg, Sertraline 100mg, neither acutely commenced |
| Outcome | Lesions resolved following cessation of Semaglutide without other treatment. |
| **Case 2** | |
| Suspected trigger | Liraglutide (Saxenda), 0.6mg once daily |
| Time from medication commencement to disease to onset | 5 days |
| Concomitant medications | Solifenacin, Lansoprazole, occasional Ibuprofen (none acutely commenced). |
| Outcome | Initially recalcitrant to multiple treatments including Omalizumab 300mg four weekly, but responded well to increased dose of Omalizumab 600mg four weekly. |

**Table S1.** Summary table of case histories.
